# Comparative Studies of Gene Expression Kinetics: Methodologies and Insights on Development and Evolution

**DOI:** 10.3389/fgene.2018.00339

**Published:** 2018-08-22

**Authors:** Tsvia Gildor, Ben-Tabou de-Leon Smadar

**Affiliations:** Department of Marine Biology, Leon H. Charney School of Marine Sciences, University of Haifa, Haifa, Israel

**Keywords:** comparative developmental biology, developmental robustness and plasticity, development and evolution, gene expression kinetics, scaling & modeling, clustering algorithm

## Abstract

Across the animal kingdom, embryos of closely related species show high morphological similarity despite genetic and environmental distances. Deciphering the molecular mechanisms that underlie morphological conservation and those that support embryonic adaptation are keys to understand developmental robustness and evolution. Comparative studies of developmental gene regulatory networks can track the genetic changes that lead to evolutionary novelties. However, these studies are limited to a relatively small set of genes and demand extensive experimental efforts. An alternative approach enabled by next-generation sequencing, is to compare the expression kinetic of large sets of genes between different species. The advantages of these comparisons are that they can be done relatively easily, for any species and they provide information of all expressed genes. The challenge in these experiments is to compare the kinetic profiles of thousands of genes between species that develop in different rates. Here we review recent comparative studies that tackled the challenges of accurate staging and large-scale analyses using different computational approaches. These studies reveal how correct temporal scaling exposes the striking conservation of developmental gene expression between morphologically similar species. Different clustering approaches are used to address various comparative questions and identify the conservation and divergence of large gene sets. We discuss the unexpected contribution of housekeeping genes to the interspecies correlations and how this contribution distorts the hourglass pattern generated by developmental genes. Overall, we demonstrate how comparative studies of gene expression kinetics can provide novel insights into the developmental constraints and plasticity that shape animal body plans.

## Introduction

Throughout metazoans, closely related species have a highly conserved body plan despite geographic distances and genetic divergence of up to 40 million years of parallel evolution [based on studies of related species of corals (Okubo et al., 2016), nematodes (Levin et al., 2012), flies (Grun et al., 2005; Kalinka et al., 2010; Kuntz and Eisen, 2014), sea urchins (Gildor and Ben-Tabou de-Leon, 2015; Malik et al., 2017), and frogs (Yanai et al., 2011)]. Within the species, embryogenesis can withstand dramatic changes in temperature (Levine et al., 2011; Runcie et al., 2012; Kuntz and Eisen, 2014), acidity (Byrne et al., 2009; Martin et al., 2011; Stumpp et al., 2012; Pespeni et al., 2013a,b; Pan et al., 2015), salinity (Shaw et al., 2014), and other environmental changes (Kitano et al., 2010). Deciphering the molecular mechanisms that underlie morphological conservation is essential for understanding developmental stability; revealing the molecular pathways that support adaptation is a key for comprehending developmental plasticity. These understandings will also help to anticipate the effect of current environmental threats such as ocean acidification and global warming on biodiversity.

Understanding the molecular mechanisms that underlie developmental robustness is quite complex as developmental progression relays on the differential expression of thousands of genes with diverse functions: regulatory, structural, metabolic, and more (Davidson, 2010; Peter and Davidson, 2011; Pan et al., 2015). If we could decipher the structure and function of entire control networks from the upstream regulatory genes to the downstream effector genes and compare these networks between different species we would better understand developmental and evolutionary processes. However, this approach is very demanding experimentally as it requires the perturbations of every gene in the network followed by a study of the morphological phenotype and molecular consequences of the perturbation. The advents in next-generation sequencing allow researchers to study and compare one aspect of developmental progression – the kinetics of gene expression, throughout all the expressed genes, between different species (Wang et al., 2009; Yanai et al., 2011; Hashimshony et al., 2014; Levin et al., 2016; Malik et al., 2017).

Experimentally, genome-wide quantification of developmental gene expression using RNA-seq can be done, relatively easily, for any species that can be cultured in the lab. Novel approaches of *de-novo* transcriptome assembly enable the initial quantitative analysis of these data even in species that do not have a reference genome (Haas et al., 2013). However, comparing the expression of thousands of genes between different species is a challenging task. The first challenge is to accurately stage the developmental rates of different species (Yanai et al., 2011; Levin et al., 2012; Gildor and Ben-Tabou de-Leon, 2015; Gildor et al., 2017), or even stage the developmental rates of the same species under various environmental conditions (Runcie et al., 2012; Kuntz and Eisen, 2014). This staging is critical for correct comparison as developmental expression patterns are extremely dynamic and rapid changes happen within short time windows (Gildor and Ben-Tabou de-Leon, 2015; Malik et al., 2017). Therefore, mismatching time points could significantly distort the expression kinetics and result with wrong estimation of the conservation of gene expression. Once the staging is done, the next challenge is to compare the expression profiles of thousands of genes with different functions and identify conservation vs. divergence in gene expression. Here we describe some quantitative approaches that we and others applied to tackle these challenges and some of the unexpected insights on developmental constraints and plasticity that were obtained.

## Scaling Developmental Rates Between Related Species

Developmental progression varies between different species due to both genetic and environmental factors (Raff, 1996). For example, increasing temperature quickens the developmental rate without morphological changes in the embryos (Yanai et al., 2011; Runcie et al., 2012; Kuntz and Eisen, 2014; Gildor and Ben-Tabou de-Leon, 2015). Hence, to study conservation and change in gene expression kinetics between different species, we first have to accurately scale the developmental rates between the species. This can be done crudely for closely related species by identifying the times where the embryos show the strongest morphological similarity. However, this approach is not quantitative and is limited to short evolutionary distances. We recently developed a simple mathematical analysis of quantitative expression data of a relatively small set of genes, to scale the developmental rates of two closely related sea urchins that separated from their common ancestor 40 million years ago (Gildor and Ben-Tabou de-Leon, 2015). We have also implemented this approach to larger evolutionary distances of up to 500 million years of parallel evolution, within the echinoderm phylum (Gildor et al., 2017).

First, we study the expression kinetics of a small set of zygotically expressed regulatory genes that are known to have a role in early development [for example, 20 genes that encode transcription factors, signaling pathways or differentiation genes (Gildor and Ben-Tabou de-Leon, 2015; Gildor et al., 2017)]. To make sure that we scale the overall developmental rate, we select genes that are expressed in different embryonic lineages. Measuring the expression kinetics could be done by QPCR or nanostring of whole embryos, as long as the spatial expression pattern of these genes is similar in the two species. At least three biological replicates are necessary since expression levels vary between batches (Gildor and Ben-Tabou de-Leon, 2015). The comparison of the initiation times of these genes between the two species is then used to scale the developmental rates (Gildor and Ben-Tabou de-Leon, 2015). For accurate fitting of gene initiation rates it is recommended that the intervals between the time points will be short enough, especially in the dynamic range (the period of gene activation). A rule of thumb is that the interval would be about a tenth of the entire developmental time studied.

**FIGURE 1:**
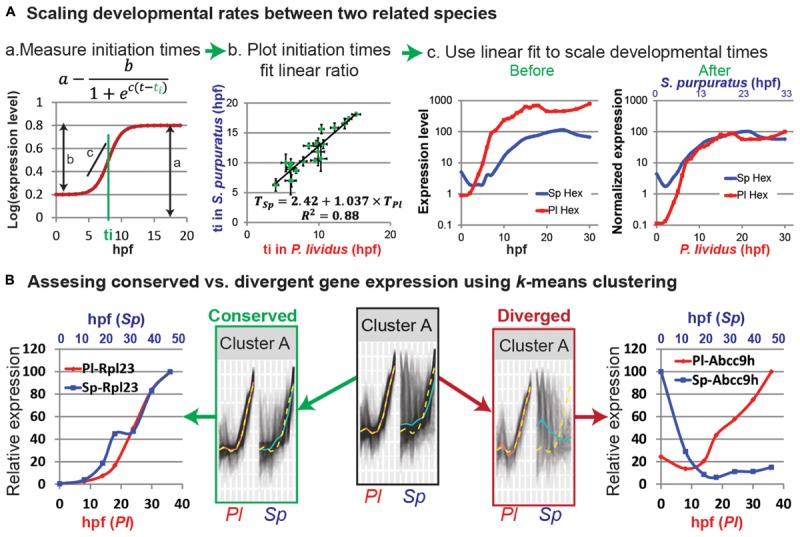
Comparative analyses of developmental expression profiles. **(A)** Scaling gene expression kinetics. **(Aa)** The initiation time, *t_i_*, of each gene in each species can be measured using a sigmoidal fit, here: *a* = 0.8; *b* = 0.6; *c* = 1 and *t_i_* = 8 hpf. **(Ab)** Gene initiation time in one species vs. initiation time in the other species. **(Ac)** The estimated linear relationship is used to scale the developmental time points in the two species. Kinetic profiles are shown before (left) and after (right) scaling and expression level normalization. **(B)** *k*-means clustering of the temporal profiles of homologous genes in *P. lividus* and *S. purpuratus*. Genes are clustered according to their expression profiles in *P. lividus* (cluster A in the middle). Yellow line indicates the cluster centroid in *P. lividus*; black lines are expression levels of genes in this cluster in *P. lividus* (left) and their orthologues in *S. purpuratus* (right); red lines and blue lines are the median of the temporal profiles of the genes in a cluster in *P. lividus* and in *S. purpuratus*, respectively. Secondly, the genes in the clusters are separated into conserved vs. diverged (see text). For example, we detect ribosomal genes in the conserved cluster (left) and ABC transporters in the diverged cluster (right).

Most zygotic genes have a clear activation curve that can be well fitted with the following sigmoidal function: $\log\left(mRNA\left(t\right)\right)=a-\frac{b}{1+e^{c\left(t-t_{i}\right)}}$ (**Figure 1Aa**; Yanai et al., 2011). Here ***a*** is the final expression level, ***b*** is the increase in level relative to the basal expression level, *c* is the slope of the curve, and ***t_i_*** is the ***initiation time***, that is, half-rise time, the time when the expression level is half of the total increase (**Figure 1Aa**). The initiation times of all measured genes in each species are estimated using this function. The initiation times in one species is then plotted relative to gene initiation times in the other species. In **Figure 1Ab** we use published measurements of the initiation times of 22 developmental genes in the sea urchins species, *Paracentrotus lividus* (*P. lividus*) and *Strongylocentrotus purpuratus* (*S. purpuratus*) (Materna et al., 2010; Gildor and Ben-Tabou de-Leon, 2015). These two species diverged from their common ancestor about 40 million years ago and are geographically separated: *S. purpuratus* occupies the west coasts of the Pacific Ocean and *P. lividus* occupies the east coasts of the Atlantic Ocean and the Mediterranean Sea. Yet, despite the genetic and geographic distance their embryonic body plan is highly similar. We measured gene initiation times in the two species based on their expression kinetics up to late gastrula stage [30 hpf in *P. lividus* and 48 hpf in *S. purpuratus* (Materna et al., 2010; Gildor and Ben-Tabou de-Leon, 2015)]. The trend-line gives the linear relationship between the developmental time in *S. purpuratus* and *P. lividus*: *T_Sp_* = 2.42 + 1.037 ×*T_Pl_*. Here the constant, 2.42, corresponds to the shift in the maternal to zygotic transition that is about two and a half hours later in *S. purpuratus* compared to *P. lividus*. The slope, 1.037, is a little higher than 1, since the developmental rate in *S. purpuratus* is slower than in *P. lividus*, possibly due to the lower culture temperature of *S. purpuratus* [15 vs. 18°C, (Gildor and Ben-Tabou de-Leon, 2015)].

Temporal scaling can now be done by matching the time points in the two species using this linear relationship. For proper comparison it is better to also normalize the expression level since different experimental parameters, like primer efficiency and measurement technique can affect the measured absolute levels, regardless of the actual differences. We normalize the expression level of each gene by dividing the level at each time point in the maximal mRNA level measured; so 100% is the maximal expression in this time interval. For example, we plot the expression kinetics before and after scaling and normalization of the regulatory gene, *hex*, in both species [**Figure 1Ac**, data from (Gildor and Ben-Tabou de-Leon, 2015)]. *hex* encodes a transcription factor that participates in the specification of the skeletogenic lineage in the sea urchin (Oliveri et al., 2008). This striking conservation of gene expression dynamics between these two closely related sea urchin species is apparent for most of the 25 developmental genes we studied (Gildor and Ben-Tabou de-Leon, 2015).

We applied this method to larger evolutionary distances, and compared developmental gene expression between sea star and sea urchin that shared a common ancestor about 500 million years ago (Gildor et al., 2017). The sea urchin and the sea star have morphological similarities as well as differences in their larval body plan and accordingly, the expression profiles of regulatory genes show mild heterochronies and loose scaling between these species. In larger evolutionary distances and when the morphologies of the embryos are critically different, it might be possible to use sets of housekeeping genes to scale the developmental rates as the developmental expression of these genes is dynamic and highly conserved between distant species (Kalinka et al., 2010; Levin et al., 2016; Malik et al., 2017). Thus, temporal scaling of developmental rates is essential for correct comparison of expression kinetics between different species and can be done based on a high-resolution quantification of the expression kinetics of a relatively small set of genes.

## Comparing the Expression Kinetics Between Thousands of Genes Using Clustering Algorithms

Comparative developmental transcriptomes provide the temporal expression kinetics of all expressed genes and by that significantly broaden the scope of the study (Kalinka et al., 2010; Yanai et al., 2011; Hashimshony et al., 2015; Israel et al., 2016; Levin et al., 2016; Malik et al., 2017). This vast scale of the transcriptomes imposes a challenge to compare the expression of thousands of genes at multiple developmental time points in different species. The use of clustering approaches enables to identify sets of genes/developmental time points that are more similar to each other than to those in other sets. These approaches can be used to answer fundamental questions such as: which time points show the highest similarity in gene expression within and between the species? Which gene sets show conserved expression pattern and which gene sets show divergence? Below we describe how different clustering analyses can be used to address these and other questions.

***Principle component analysis (PCA)*** provides a computational way to identify the experimental time points in different species that show the highest similarity in gene expression, that is, it enables **sample grouping** (Ringner, 2008). The algorithm identifies directions, called principal components, along which the variation in gene expression throughout the samples, is maximal. Then it uses these components to represent each sample by relatively few numbers instead of by the expression level of thousands of genes. Samples can then be plotted, making it possible to visually assess similarities and differences between experimental time points. This method could be used to guarantee that indeed the temporal staging is accurate and equivalent points in the different species map together (Levin et al., 2012; Israel et al., 2016; Malik et al., 2017). When more than two species are compared, this analysis can be applied to test which species have higher similarity in their gene expression (Israel et al., 2016).

Other approaches **group genes together** according to the similarity in their expression profiles: ***Hierarchical clustering*** uses standard statistical algorithms to group together genes that have similar expression patterns (Eisen et al., 1998). This approach is very informative regarding the different expression profiles that exist in developing embryos (Gildor et al., 2016; Levin et al., 2016). ***K-means clustering*** partitions the expression profiles of thousands of genes into k clusters in which each gene belongs to the cluster with the nearest mean (Do and Choi, 2008). The clustering algorithm starts by randomly choosing k expression patterns as initial means for each cluster. Then, each gene is assigned to the cluster that has the closest mean to the gene expression pattern. The new mean is calculated for each cluster and the genes are partitioned again according to the new means. This process is repeated until the cluster means are such that no gene moves from one cluster to another. This analysis results with a partition of all expressed genes into groups of genes that have a similar expression profile, represented by the cluster mean. Both Hierarchical and K-means clustering facilitate the use of gene ontology (GO) enrichment tools to identify functional classes within the clusters, which is very helpful in relating gene expression patterns to gene function (Malik et al., 2017).

*k*-means can also be used to identify interspecies conservation/divergence in the expression of orthologous genes across several related species. Genes are first clustered according to their expression kinetics in one species and then this clustering is used to identify conservation or change in their orthologues gene expression in other species (**Figure 1B**; Israel et al., 2016; Malik et al., 2017). Conservation can be defined as the mapping of a gene to the same cluster in multiple species and divergence as the mapping of a gene to different clusters in different species (Israel et al., 2016). It can also be defined by the distance of a gene expression profile in one species from the mean of the cluster of its orthologue in another species, as we exemplify in **Figure 1B** (Malik et al., 2017). Here we used *k*-means to compare the developmental transcriptomes measured at seven equivalent developmental time points in *P. lividus* (between 0 and 36 hpf) and *S. purpuratus* (between 0 and 48 hpf) (Tu et al., 2012; Malik et al., 2017). First *k*-means clustering was used to group genes that have similar expression kinetics in *P. lividus*, into 10 clusters (for example, cluster A, **Figure 1B**). A gene was identified as conserved if the Euclidian distance of its expression profile in *S. purpuratus* from its *P. lividus* cluster-centroid was shorter than a certain cutoff and diverged otherwise (conserved/diverged cluster A in **Figure 1B**; Malik et al., 2017). The cutoff was defined based on the maximal distances of *P. lividus* gene expression from the *P. lividus* centroids (Malik et al., 2017). We then used various GO-enrichment tools to identify functional classes that were over-represented in each conserved or diverged cluster. We discovered that the developmental expression of common housekeeping genes is dynamic and highly conserved between the two species, probably due to developmental constraints on these genes. For example, the expression of ribosomal genes is highly conserved and increases throughout development, possibly to provide the growing embryo with sufficient translation machinery (*e.g.*, *rpl23* in conserved cluster A, **Figure 1B**). On the other hand we discovered that some homeostasis and environmental response genes show diverged expression, either due to evolutionary drift or to allow for adaptation to the local environmental conditions (*e.g.*, the ATP-potassium channel, *abcc9h* in diverged cluster A, **Figure 1B**). Thus, *k*-means clustering is a useful tool to identify typical gene expression patterns, test for interspecies conservation and identify the functional gene classes that show this typical expression pattern.

## The Interspecies Correlations of Housekeeping Genes Has a Funnel Shape That Distorts the Developmental Hourglass Pattern

A fundamental quest in evolution and development is to identify the ***phylotypic stage***, a stage in which developing embryos of species in the same phylum display maximal morphological similarity (Raff, 1996). If such a stage exists in different phyla, identifying it could illuminate the developmental bottlenecks that constraint the evolution of body plans (Slack et al., 1993). Furthermore, this would indicate that evolutionary processes within a phylum are somehow distinct form evolutionary processes between phyla (Hejnol and Dunn, 2016).

The *phylotypic stage* concept was based on comparative morphological studies of selected vertebrates’ embryos by Haeckel (Haeckel, 1874) and Baer (von Baer, 1828), that showed high similarity at the tailbud stage (Slack et al., 1993). These and other studies gave rise to two conservation models; the **funnel** model assumes the highest conservation at early embryonic stages whereas the ***hourglass model*** suggests that intermediate developmental stages are most resistant to evolutionary changes (Slack et al., 1993; Raff, 1996). Later morphological studies, that extended Baer and Haeckel comparison to larger sets of vertebrates’ embryos, showed considerable variability in multiple morphological characteristics during the tailbud stage (Richardson et al., 1997). The advent of next generation sequencing provided the opportunity to resolve this debate by searching for a developmental time that shows the highest interspecies conservation of gene expression that could be defined as the molecular phylotypic stage.

Different computational approaches were used to assess the level of interspecies conservation of gene expression. A method that is commonly applied for this measurement is the Pearson or Spearman correlations between gene expression in equivalent time points of two different species (Irie and Kuratani, 2011; Yanai et al., 2011; Levin et al., 2016; Malik et al., 2017). ***Pearson correlation*** measures the linear relationship between two variables while ***Spearman correlation*** measures how well the relationship between two variables can be described as a monotonic function, even if it is not linear. In both methods, the higher the correlation, the stronger the conservation of the ratios between the expression levels of different genes. The advantage of these pair-wise comparisons is that they are relatively simple to use and intuitive to interpret. However, when interpolating the results of multiple pair-wise comparisons to assess the conservation patterns between various species, the phylogenetic distances should be explicitly considered, otherwise distant species could lead to a pattern distortion (Dunn et al., 2018). Other methods compute the interspecies divergence in gene expression as the variance in gene expression in each time point across different species (Kalinka et al., 2010; Yanai et al., 2011). These different approaches were used to quantify the interspecies conservation in gene expression between various species across the animal kingdom.

The relationship between gene expression conservation and the morphological phylotypic stage seem to be quite complex. The conservation in gene expression between equivalent time points of two closely related *Xenopus* species increases with developmental progression, even after the morphological phylotypic stage (Yanai et al., 2011). However, the Spearman correlation of gene expression between four vertebrates’ species were the highest at the mid-developmental stage, in agreement with Haeckel and Baer observation of the morphological phylotypic stage in the vertebrate phylum (Irie and Kuratani, 2011). Relatedly, the interspecies divergence of gene expression between six *Drosophila* species is the lowest at mid-development, which could indicate that this developmental stage is the phylotypic stage in flies (Kalinka et al., 2010). These vertebrates and drosophila studies support the hourglass model of conservation within a phylum. A recent comparative study of 10 species representing 10 different phyla shows that mid-developmental transition corresponds to the time of lowest interspecies correlations between the phyla (Levin et al., 2016). Thus, intriguingly, the time of highest conservation in gene expression within the phylum is the time of lowest conservation between different phyla (Levin et al., 2016). This conclusion was criticized due to the inclusion of a distant species (ctenophores) that distorts the conservation pattern (Dunn et al., 2018). Regardless of methodological disagreements, the relationship between interspecies conservation of gene expression and the interspecies morphological similarity or divergence is still not clear.

**FIGURE 2:**
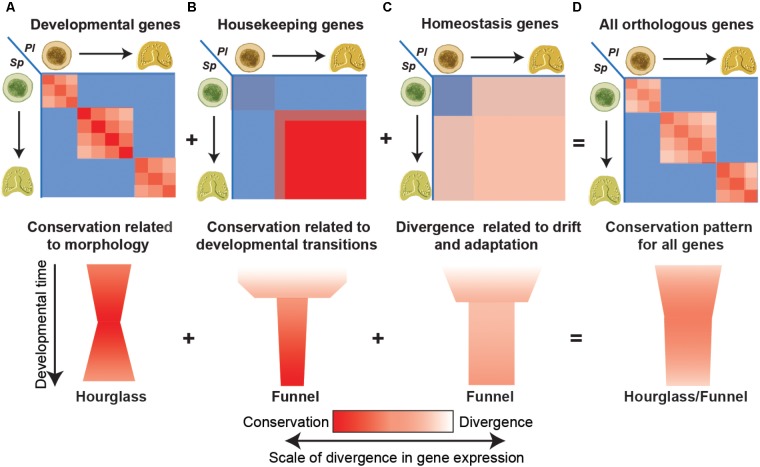
Different functional classes have different interspecies conservation patterns. **(A)** The interspecies correlations between genes with developmental GO-terms are strongest between equivalent time points and pick at intermediate developmental times (upper panel) supporting the hourglass model (bottom panel). **(B)** The interspecies correlation of different housekeeping genes rapidly increases after the maternal to zygotic transition and increases with developmental progression giving rise to a funnel pattern of conservation. **(C)** The interspecies correlation of homeostasis and environmental response genes are intermediate throughout development regardless of morphological similarity resulting in a broad funnel conservation pattern. **(D)** When all genes are combined the different contributions merge, and the hourglass pattern is superimposed with the funnel pattern.

We believe that some of the conflicting observations could result from the different contribution of different functional classes to the pattern of gene expression conservation and how these contributions change with increasing evolutionary distances (**Figure 2**; Malik et al., 2017). Comparing gene expression between two closely related sea urchin species we noticed that the conservation of developmental gene expression is tightly related to morphological similarity and therefore is diagonal and picks at equivalent developmental stages (**Figure 2A**). Thus, within the phylum the conservation of these genes is highest at mid-development, resulting with an hourglass pattern (**Figure 2A**). On the other hand, the conservation of certain sets of housekeeping genes like ribosomal genes, RNA-processing genes and mitochondrial genes was relatively low at early time points and significantly increased after the maternal to zygotic transition (**Figure 2B**; Malik et al., 2017). Importantly, within these sets, high interspecies correlation was observed between all zygotic time points, regardless of morphological similarity and the correlation increases with developmental time. Other sets of genes with GO terms related to homeostasis and environmental response show intermediate levels of interspecies correlation between morphologically similar and dissimilar time points, possibly reflecting evolutionary drift or adaptation (**Figure 2C**; Malik et al., 2017). When all expressed genes are considered together, the different conservation patterns merge into a hourglass/funnel shape (**Figure 2D**; Malik et al., 2017).

With increasing evolutionary distances and diverged morphologies we expect that developmental gene expression would be less conserved and will contribute less to the interspecies correlation patterns. On the other hand, the expression dynamics of housekeeping genes is related to universal embryonic transitions, such as the maternal to zygotic transition or gastrulation that demand a boost in the expression of these genes. Therefore, housekeeping expression dynamics in development is expected to be highly conserved, even between highly divergent species. Thus, at high evolutionary distances, the main contribution to the conservation pattern could come from the housekeeping genes and reflect major embryonic transitions and not morphological similarity vs. divergence. Thus to better understand the evolutionary constraints of gene expression and identify a molecular phylotypic stage, the interspecies correlation should be done separately to different classes of genes.

## Conclusion

Large-scale comparative analyses of developmental gene expression inflict a great computational challenge that requires the use of appropriate methods. Novel insights on developmental constraints and flexibility can be gained from a careful analysis of the data. Attention should be given to the dynamic expression of genes that are not considered developmental and seem to evolve under a different set of constraints that is not tightly related to morphology and could be very influential with increasing evolutionary distances.

## Author Contributions

Both authors have made a substantial, direct and intellectual contribution to the work, and approved it for publication.

## Conflict of Interest Statement

The authors declare that the research was conducted in the absence of any commercial or financial relationships that could be construed as a potential conflict of interest.
