# Corrigendum: Comparative Studies of Gene Expression Kinetics: Methodologies and Insights on Development and Evolution

**DOI:** 10.3389/fgene.2018.00631

**Published:** 2018-12-07

**Authors:** Tsvia Gildor, Smadar Ben-Tabou de-Leon

**Affiliations:** Department of Marine Biology, Leon H. Charney School of Marine Sciences, University of Haifa, Haifa, Israel

**Keywords:** comparative developmental biology, developmental robustness and plasticity, development and evolution, gene expression kinetics, scaling & modeling, clustering algorithm

In the original article, there was a mistake in Figure 1 as published. There is a mistake in the formulas in Figures 1Aa,b. The corrected Figure 1 appears below.

**Figure 1:**
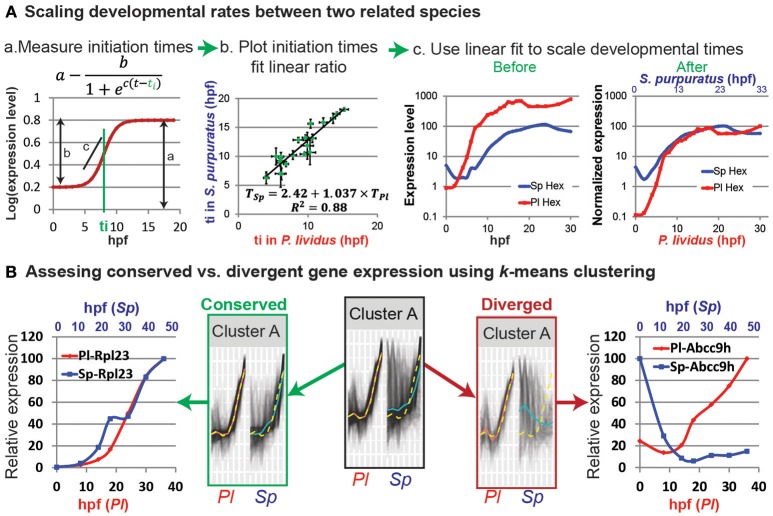
Comparative analyses of developmental expression profiles. **(A)** Scaling gene expression kinetics. **(Aa)** The initiation time, *t*_*i*_, of each gene in each species can be measured using a sigmoidal fit, here: *a* = 0.8; *b* = 0.6; *c* = 1 and *t*_*i*_ = 8 hpf. **(Ab)** Gene initiation time in one species vs. initiation time in the other species. **(Ac)** The estimated linear relationship is used to scale the developmental time points in the two species. Kinetic profiles are shown before (left) and after (right) scaling and expression level normalization. **(B)** *k*-means clustering of the temporal profiles of homologous genes in *P. lividus* and *S. purpuratus*. Genes are clustered according to their expression profiles in *P. lividus* (cluster A in the middle). Yellow line indicates the cluster centroid in *P. lividus*; black lines are expression levels of genes in this cluster in *P. lividus* (left) and their orthologues in *S. purpuratus* (right); red lines and blue lines are the median of the temporal profiles of the genes in a cluster in *P. lividus* and in *S. purpuratus*, respectively. Secondly, the genes in the clusters are separated into conserved vs. diverged (see text). For example, we detect ribosomal genes in the conserved cluster (left) and ABC transporters in the diverged cluster (right).

In the original article, there was an error. There is a mistake in the sigmoid formula, we wrote $\log\left(mRNA\left(t\right)\right)=a-\frac{b}{e^{c\left(t-t_{i}\right)}}$ and the correct formula is: $\log\left(mRNA\left(t\right)\right)=a-\frac{b}{1+e^{c\left(t-t_{i}\right)}}$

A correction has been made to the section Scaling developmental rates between related species, Paragraph Number 3:

Most zygotic genes have a clear activation curve that can be well fitted with the following sigmoidal function: $\log\left(mRNA\left(t\right)\right)=a-\frac{b}{1+e^{c\left(t-t_{i}\right)}}$ (Figure 1Aa; Yanai et al., 2011). Here ***a*** is the final expression level, ***b*** is the increase in level relative to the basal expression level, ***c*** is the slope of the curve, and ***t***_***i***_ is the initiation time, that is, half-rise time, the time when the expression level is half of the total increase (Figure 1Aa). The initiation times of all measured genes in each species are estimated using this function. The initiation times in one species is then plotted relative to gene initiation times in the other species. In Figure 1Ab we use published measurements of the initiation times of 22 developmental genes in the sea urchins species, *Paracentrotus lividus* (*P. lividus*) and *Strongylocentrotus purpuratus* (*S. purpuratus*) (Materna et al., 2010; Gildor and Ben-Tabou de-Leon, 2015). These two species diverged from their common ancestor about 40 million years ago and are geographically separated: *S. purpuratus* occupies the west coasts of the Pacific Ocean and *P. lividus* occupies the east coasts of the Atlantic Ocean and the Mediterranean Sea. Yet, despite the genetic and geographic distance their embryonic body plan is highly similar. We measured gene initiation times in the two species based on their expression kinetics up to late gastrula stage [30 hpf in *P. lividus* and 48 hpf in *S. purpuratus* (Materna et al., 2010; Gildor and Ben-Tabou de-Leon, 2015)]. The trend-line gives the linear relationship between the developmental time in S. purpuratus and *P. lividus*: *T*_*Sp*_ = 2.42+1.037 × *T*_*Pl*_. Here the constant, 2.42, corresponds to the shift in the maternal to zygotic transition that is about two and a half hours later in S. purpuratus compared to *P. lividus*. The slope, 1.037, is a little higher than 1, since the developmental rate in *S. purpuratus* is slower than in *P. lividus*, possibly due to the lower culture temperature of *S. purpuratus* [15 vs. 18°, (Gildor and Ben-Tabou de-Leon, 2015)].

The authors apologize for this error and state that this does not change the scientific conclusions of the article in any way. The original article has been updated.

## Conflict of Interest Statement

The authors declare that the research was conducted in the absence of any commercial or financial relationships that could be construed as a potential conflict of interest.
